# Supplementary material for: Reduced Plasma Guanylin Levels Following Enterotoxigenic Escherichia coli-Induced Diarrhea
Source: Microorganisms. 2023 Aug 3;11(8):1997. doi: 10.3390/microorganisms11081997 (PMC10458898; doi:10.3390/microorganisms11081997)
Supplement: Supplementary file 1 [file microorganisms-11-01997-s001.zip › microorganisms-2496545-supplementary.pdf]

Supplementary figures and tables

Article

Plasma guanylins and urine electrolytes during human experimental ETEC infection

Ingeborg Brønstad, Hilde Løland von Volkmann, Sunniva Todnem Sakkestad, Hans Steinsland and Kurt Hanevik

Supplementary Figure S1. Correlations between changes in proguanylin (ΔproGN) and prouroguanylin (ΔproUGN) and 24 hours total stool weight (both diarrheal and non-diarrheal stools) before blood sampling. For the diarrhea group the 24 hours before blood sampling that included the main part of the diarrhea episode was used. For comparison also data for the non-diarrhea group the 24-hour period before blood sampling on day 2 is included in the figure.
Spearman test (including only diarrhea cases) for ΔproGN vs stool weight: *r*=-0.249, *p*=0.484
Spearman test (including only diarrhea cases) for ΔproUGN vs stool weight: *r*=0.561 *p*=0.096
Open circles: non-diarrhea group, solid circles: diarrhea group. Red circles: severe diarrhea. Green circles: moderate diarrhea. Blue circles: mild diarrhea.

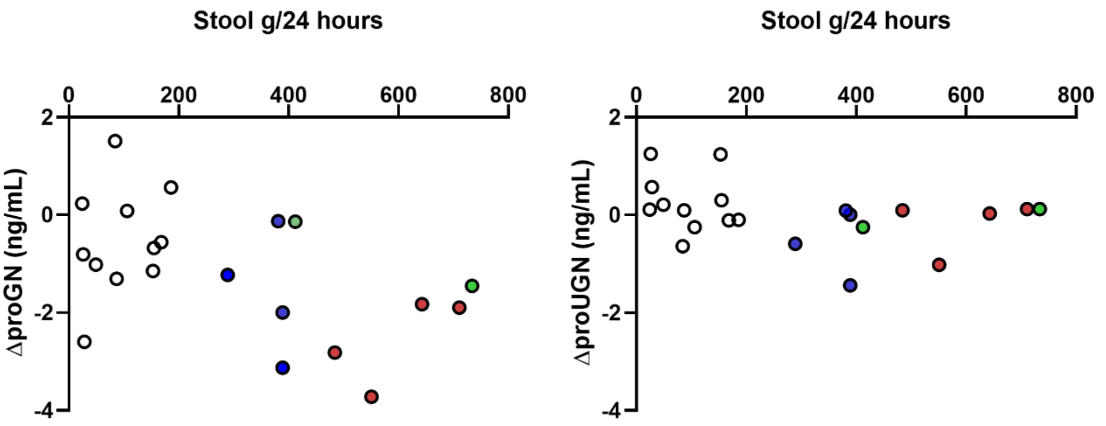

Supplementary Table S1. Median values (25th, 75th percentiles) of plasma proguanylin (proGN) and prouroguanylin (proUGN) in healthy volunteers with or without diarrhea, before (day 0) and 1, 2, 3 and 7 days after ETEC ingestion.

	Diarrhea (n = 10)	Non-diarrhea (n = 11)	*p* - value
pro-guanylin (ng/mL)			
Day 0	7.20 (6.45, 7.78)	6.61 (6.46, 7.27)	0.426
Day 1	7.07 (6.64, 8.39)	6.83 (6.50, 9.17)	0.667
Day 2	5.71 (5.21, 6.39)	5.90 (5.69, 7.06)	0.075
Day 3	5.64 (4.95, 6.34)	6.49 (6.20, 7.37)	**0.005**
Day 7	6.59 (5.14, 6.99)	6.53 (6.14, 7.39)	0.654
pro-uroguanylin (ng/mL)			
Day 0	2.09 (1.79, 2.24)	2.08 (1.66, 2.51)	0.932
Day 1	2.17 (1.94, 2.38)	2.17 (1.55, 2.50)	0.958
Day 2	1.68 (1.46, 2.12)	1.87 (1.36, 2.09)	0.986
Day 3	1.71 (1.53, 2.04)	1.87 (1.34, 2.20)	0.640
Day 7	1.80 (1.56, 2.02)	1.99 (1.59, 2.60)	0.358

p-value calculated by Mann-Whitney tests between the diarrhea and non-diarrhea groups for each day.

Supplementary Table S2. Correlations between plasma proGN and proUGN in healthy volunteers with or without diarrhea, before (day 0) and 1, 2, 3 and 7 days after ETEC ingestion.

	Diarrhea		Non-diarrhea	
Day	*r*	*p*	*r*	*p*
0	0.697	0.031	0.618	0.048
1	0.742	0.018	0.518	0.107
2	0.058	0.874	0.755	0.010
3	0.110	0.762	0.455	0.163
7	0.467	0.179	0.400	0.225
